# A prospective cohort study on lactation status and breastfeeding challenges in mothers giving birth to preterm infants

**DOI:** 10.1186/s13006-021-00447-4

**Published:** 2022-01-10

**Authors:** Dingding Dong, Xifang Ru, Xiaofang Huang, Tian Sang, Shan Li, Ying Wang, Qi Feng

**Affiliations:** Department of Pediatrics, Peking University First Hospital, No.1 Xi’anmen Street, Xicheng District, Beijing, China

**Keywords:** Infant, Premature, Breastfeeding, Lactogenesis II

## Abstract

**Background:**

Mothers of preterm infants face many challenges in breastfeeding, especially regarding lactation. This study aimed to investigate the lactation status and challenges in breastfeeding faced by preterm infants’ mothers.

**Methods:**

We approached 124 mothers who gave birth to preterm infants between 26 May and 31 October 2018 in a tertiary hospital in China. Lactation status and challenges in breastfeeding on day 7 postpartum, at discharge of infants, 2 weeks post-discharge, and 3 months of corrected age were collected using questionnaires. The area under the receiver operating characteristic (ROC) curve for expressed milk volume on day 7 postpartum for predicting expressed milk volume ≥ 300 mL/d at discharge was calculated. Logistic regression analyses were performed to identify factors associated with delayed lactogenesis II onset and continuation of breastfeeding at 3 months of corrected age.

**Results:**

Seventy mothers were enrolled, and 51.4% had delayed lactogenesis II. Multivariate logistic regression analysis revealed that older maternal age (aOR = 1.19; 95% CI: 1.01, 1.40) and first live birth (aOR = 4.81; 95% CI 1.43, 16.18) were significant independent predictors of delayed lactogenesis II. Mothers with delayed lactogenesis II had significantly lower expressed milk volume (day 7 postpartum: 160.0 mL vs. 300.0 mL, *U* = 328.50, *p* = 0.001; at discharge: 425.0 mL vs. 612.5 mL, *U* = 372.00, *p* = 0.005), with a lower proportion of exclusive breastfeeding in their infants (at discharge: 33.3% vs. 69.8%, *χ*^*2*^ = 12.39, df = 1, *p* < 0.001; 3 months of corrected age: 17.8% vs. 52.8%, *χ*^*2*^ = 11.03, df = 1, *p* = 0.001). The ROC showed that expressed milk volume > 190 mL/d on day 7 postpartum significantly predicted expressed milk volume ≥ 300 mL/d at discharge. Insufficient human milk was the main reason for breastfeeding discontinuation at 3 months of corrected age. Twins were less likely to continue breastfeeding at 3 months of corrected age (aOR = 0.27; 95% CI 0.09, 0.86). In singleton infants, mother’s own milk ≥50% of total milk uptake at 2 weeks post-discharge (aOR = 32.66; 95% CI 3.00, 355.25) was an independent predictor of continuous breastfeeding at 3 months of corrected age. Feeding complications in infants, poor breastfeeding technique, and low milk output are the main challenges in breastfeeding.

**Conclusion:**

Interventions to improve early postpartum lactation and breastfeeding techniques may increase breastfeeding adoption in mothers of preterm infants.

**Supplementary Information:**

The online version contains supplementary material available at 10.1186/s13006-021-00447-4.

## Background

Preterm infants (gestational age [GA] < 37 weeks) benefit from breastfeeding in many ways, including shorter hospital stay [1–3], lower incidence of severe complications including necrotizing enterocolitis [3–5] and bronchopulmonary dysplasia [2], better developmental outcomes, and lower incidence of rehospitalization [6]. Therefore, fortified human milk has been recognized as an ideal food for preterm infants in neonatal intensive care units (NICUs) [7].

However, the prevalence of breastfeeding among preterm infants varies greatly worldwide. In the United States, 75% of preterm infants in NICUs were breastfed in 2015 [8]. In Europe, 58.5% of preterm infants (GA < 32 weeks) received human milk at discharge, and 46.3% of the NICUs used donor human milk to feed very preterm infants whose mothers’ milk was not available [9]. In China, the prevalence of breastfeeding in preterm infants (GA < 37 weeks) varies widely among hospitals [10]. Hei et al. investigated the preterm infants (GA 28–35 weeks) in 11 NICUs in China and found a predominantly breastfeeding (breast milk > 1/2 the daily feeding volume) prevalence of only 55.78% in 2017 [11]. Another study involving 24,113 preterm infants (GA < 34 weeks) in China in 2018 indicated that preterm infants’ breastfeeding and exclusive breastfeeding rates were 58.2 and 18.8%, respectively [12]. In Nanjing Maternity and Child Health Care Hospital, donor human milk from a human milk bank was available for all infants with very low or extremely low birth weight [13].

Parents usually participate in breastfeeding education and training during pregnancy, however, the stress associated with preterm delivery often disrupts their original breastfeeding plan [14]. Although the breastfeeding of preterm infants has significantly improved in recent years [12, 15–17], it remains a big challenge for mothers who give birth to sick infants and thus have to be separated from their infants in the NICU. In addition, in most NICUs in China, mothers are frequently unable to visit their infants [11, 18]. Preterm infants admitted to the NICU often have critical conditions and low birth weight, and human milk, especially their mothers’ milk, is definitively the best food for them. As human milk bank has not been commonly utilized in China currently [19], preterm infants in China mostly rely on their own mother’s milk. To support breastfeeding, hospitals encourage mothers to feed their preterm infants with their own expressed milk, which can be frequently sent to the NICU.

The onset of lactogenesis II, which is defined as the time when mothers experience a sudden increase in the amount of breast milk, is an important indicator of early lactation [20]. In previous studies, the onset of lactogenesis was measured by testing infants’ weight gain before and after feeding; recording mothers’ perception of milk “coming in” [20]; measuring changes in citrate, lactose, sodium, and total protein [21]; and measuring the change in expressed milk volume [22]. Commonly, it occurs at 50–73 h postpartum [20, 23], and delayed lactogenesis II onset is defined as failure to occur within 72 h [20]. In mothers of preterm infants, delayed lactogenesis II onset is more common [24, 25]. Delayed lactogenesis II onset can adversely affect breastfeeding in later periods. Previous studies found that mothers with delayed lactogenesis II onset had lower expressed milk volume on day 7 postpartum than mothers without delayed lactogenesis II onset [26] and that mothers with delayed lactogenesis II onset were less likely to exclusively breastfeed at 4 weeks postpartum [27]. Delayed lactogenesis II onset was also found to be associated with premature discontinuation of breastfeeding [28, 29].

While there is still room for improving the breastfeeding of preterm infants, studies on lactation status and the challenges of different breastfeeding stages facing the mothers of preterm infants in China are limited. Therefore, we aimed to investigate lactation status and understand the challenges in breastfeeding faced by mothers giving birth to preterm infants, to develop effective interventions to improve breastfeeding in preterm infants in the NICU in the future.

## Methods

### Study design and setting

This was a prospective cohort study. The study protocol was approved by the institutional review board of the hospital (project identification code: 2018–88; date of approval: May 23rd, 2018). Written informed consent was obtained from all participating mothers.

### Study participant inclusion and exclusion criteria

Mothers of preterm infants were recruited between May 26th and October 31st, 2018 on day 1 to day 4 of infants’ NICU entry, and were followed up until infants’ 3 months of corrected age. The inclusion criteria were: (i) mothers giving birth to infants before 37 weeks of gestation and their infants being admitted to the NICU and (ii) mothers intended to breastfeed their infants. The exclusion criteria were as follows: (i) mothers did not sign the informed consent form, (ii) infant death, (iii) infants transferred to another department, and (iv) mothers took their infants home against medical advice.

### Infant feeding procedure

Enteral feeding was started as soon as the infant’s condition became stable and there were no contraindications (digestive tract malformation or hemodynamic instability). In infants with neonatal asphyxia, enteral feeding was started 12–24 h postpartum [30]. The expressed own mothers’ milk was prioritized [7]. The mothers were encouraged to express milk postpartum as soon as their conditions became stable, and their expressed milk was sent to the NICU directly while the mothers stayed in the obstetric ward. After the mothers were discharged from the hospital, their expressed milk was frozen at home and then transported to the NICU by their family members. The infants were fed with their own mothers’ milk via either a gastric tube or bottle during their hospital stay by healthcare staff. Human milk fortifier (HMF) was added in infants with a GA < 34 weeks or birth weight < 2000 g [30]. HMF was initiated when the total volume of enteral feeding reached 50 mL/kg/d [31] and their own mothers’ milk was available. There were two levels of standardized fortified human milk with commercial fortifier in our NICU: full strength fortified human milk (80–85 kcal and 2.5–3.0 g protein/100 mL) for infants with birth weight < 1800 g or who were fluid-restricted or failing to thrive, and half-strength fortified human milk (74 kcal and 2.0 g protein/100 mL) provided at ≥150 mL/kg/day [32, 33]. Infants fed full-strength fortified human milk were started on half-strength and gradually advanced to full-strength level after 48 h [34]. All feeding information was documented daily in the medical records, including feeding volume, human milk volume, and tolerance. Direct breastfeeding was recommended after the infant no longer needed full strength HMF after discharge. If own mothers’ milk was insufficient, infants were fed with an additional preterm formula or post-discharge formula [17]. Infants were discharged if they matured, reached corrected GA ≥34 weeks, had a bodyweight ≥2000 g, had a daily milk intake volume ≥ 150 mL/kg/d, were able to suck and drink all milk orally, and had stable vital signs. The mothers and their infants were required to return for regular post-discharge follow-up examination until 2 years of corrected age. Infants’ information, including perinatal data, feeding, and medical conditions in hospital and follow-up clinics, was collected from the hospital’s electronic medical record systems.

### Questionnaire survey for participating mothers

Participating mothers were required to complete questionnaires on lactation and breastfeeding practice on day 7 postpartum, the day of infant discharge, 2 weeks after infant discharge, and 3 months corrected age of infants either by filling the questionnaires by themselves or in telephone conversations (Additional File 1).

In general, the questionnaires collected mothers’ input on expressing milk practice, expressed milk volume, and challenges in breastfeeding. In particular, on day 7 postpartum, the questionnaire collected information regarding expressed milk volume in the past 24 h, the type of pump used, milk expression frequency, and day of lactogenesis II onset. This time point showed the status of breastfeeding initiation in mothers of preterm infants. At the discharge of infants, the questionnaire, which was completed within 48 h before discharge, collected data on expressed milk volume in the past 24 h, milk expression frequency, and challenges in breastfeeding during the infant hospital stay. The questionnaire that was conducted 2 weeks after infants’ discharge and at 3 months of corrected age collected infants’ feeding method, mothers’ perception of milk volume changes, reasons for discontinuing breastfeeding, and challenges in breastfeeding at home. Two weeks post-discharge was the first time point in regular follow-up clinics in our hospital, therefore, it could demonstrate the challenges parents faced when feeding their infants independently shortly after discharge. In our regular follow-up clinics, the next time point after 3 months of corrected age was 4–6 months of corrected age. It is recommended that complementary food be introduced between 4 and 6 months in term infants [35], and preterm infants usually introduce complementary food between 4 and 6 months of corrected age. Therefore, we used 3 months of corrected age as the time point to check breastfeeding status relatively long-term post-discharge and prior to complementary food introduction.

### Definitions

Breastfeeding was defined as feeding infants either partially or exclusively human milk for 24 h at each time point. Human milk volume was defined as the volume of human milk in 24 h. Human milk proportion was the proportion of human milk in the total milk consumed by infants in 24 h [36]. Expressed milk volume was the total expressed human milk volume in 24 h [37]. Lactogenesis II onset was defined as the day when mothers were able to express 20 mL milk (the total amount from both sides) for three consecutive pump sessions [22]. Delayed lactogenesis II onset is usually defined as failing to appear within 72 h postpartum [20], and is sometimes adapted to 4 days in clinical research [27]. Therefore, in this study, we defined delayed lactogenesis II onset as failure to occur within 4 days postpartum. Direct feeding was defined as infants who were fed directly at the breast either exclusively, or combined with bottle-feeding [38]. Feeding complications included abdominal distension, vomiting, diarrhea, hematochezia, and poor weight gain. First live birth delivery was defined as the first birth of one or more live infants, with the delivery of multiple infants counting as one single live birth delivery [39]. Live birth was defined as the delivery of a live fetus after 20 completed weeks of gestation [40].

### Statistical analysis

Non-normally distributed continuous variables are presented as median (interquartile range [IQR]). Categorical variables are presented as the number of cases and percentages. Chi-squared and Mann–Whitney U tests were performed to compare variables between different groups and analyze independent covariates. Mantel-Haenszel tests (Linear by Linear Test) were performed to compare the breastfeeding status at discharge, at 2 weeks post-discharge, and at 3 months of corrected age. The receiver operating characteristic (ROC) curve was used to examine the predictive validity of expressed milk volume on day 7 postpartum. The area under the graph was assessed for the sensitivity and specificity of expressed milk volume on day 7 postpartum in predicting expressed milk volume ≥ 300 mL/d at discharge (the estimated infants’ daily milk volume intake at discharge, 150 ml/kg/d × 2000 g = 300 ml/d).

Logistic regression analyses were performed to identify factors associated with delayed lactogenesis II onset and the continuation of breastfeeding at 3 months of corrected age. In the logistic regression analysis of factors associated with delayed lactogenesis II onset, cesarean section, maternal age, first live birth, twin, pregnancy complications, and GA were included as covariates and delayed lactogenesis II onset as the dependent variable. Maternal age was included as a continuous variable in statistical analysis. A backward LR variable selection method was used to identify significant variables among cesarean section, maternal age, first live birth, pregnancy complications, and GA. The inclusion and removal of variables in models were based on *p* values of 0.05 and 0.10, respectively. Twin was entered into the model through enter method due to potential clinical relevance. Cesarean section, older maternal age, first live birth, and twin entered the final model.

In the logistic regression analysis of continuation of breastfeeding at 3 months of corrected age, breastfeeding at 3 months of corrected age was included as a dependent variable. In the model for all infants, a backward LR variable selection method was used to identify significant variables among cesarean section, maternal age, twin, GA, exclusive breastfeeding at discharge, own mother’s milk ≥50% at 2 weeks post-discharge, and direct feeding at 2 weeks post-discharge. Twin and own mother’s milk ≥50% at 2 weeks post-discharge entered the model. In models for twins and singleton infants respectively, a backward LR variable selection method was used to identify significant variables among cesarean section, maternal age, GA, exclusive breastfeeding at discharge, own mother’s milk ≥50% at 2 weeks post-discharge, and direct feeding at 2 weeks post-discharge. The inclusion and removal of variables in models were based on *p* values of 0.05 and 0.10, respectively. Hosmer-Lemeshow test was used to assess the goodness-of-fit of the final models (*p* > 0.05).

The statistical significance level was set at *p* < 0.05. IBM SPSS Statistics (version 22.0; SPSS, Chicago, IL, USA) was used for all statistical analyses.

## Results

### Participant flow chart and clinical characteristics

**Fig. 1:**
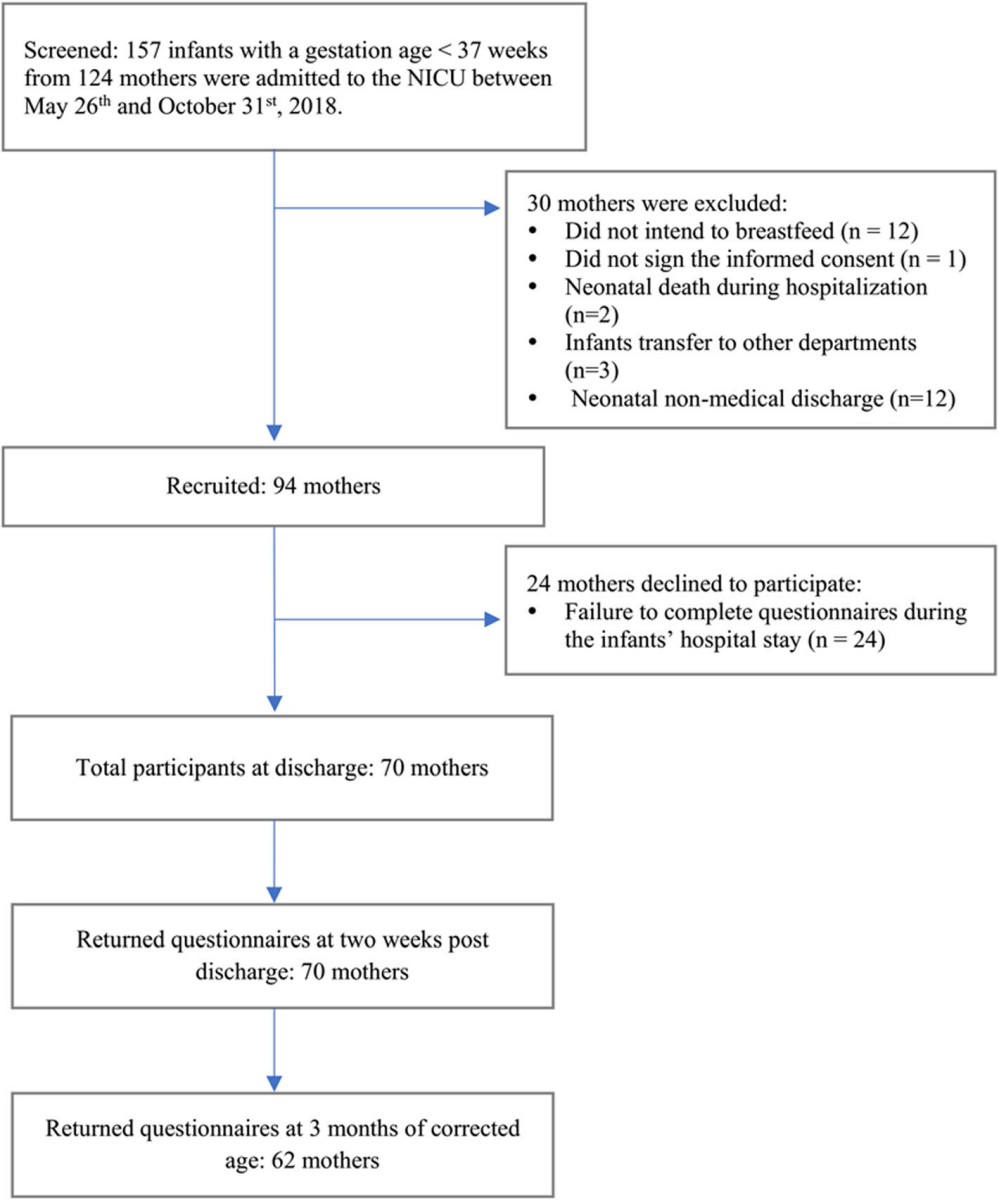
Flow chart of study participants

The participant flowchart is shown in Fig. 1. A total of 157 preterm infants with a GA < 37 weeks from 124 mothers were admitted to the NICU between May 26th and October 31st, 2018. Of the 124 mothers, 30 were excluded for the following reasons: did not intend to breastfeed (*n* = 12), did not sign the informed consent form (*n* = 1), neonatal death (*n* = 2), infants transferred to another department (*n* = 3), or neonatal non-medical discharge (*n* = 12). A total of 94 eligible mothers (full analysis set) participated in this study. During the study, 24 mothers failed to complete the questionnaire during their hospital stay. Data from 70 mothers (per protocol set) were analyzed. All 70 mothers and their 94 infants participated in the follow-up examination at 2 weeks post-discharge, while 62 mothers and their 81 infants participated at 3 months of corrected age.

Table 1 shows the clinical characteristics of mothers. The median age of the 70 mothers was 34.0 (31.0, 36.0) years. Of the 70 mothers, 64.3% (45/70) were primiparous; 62.9% (44/70) had pregnancy complications, including hypertensive disorders of pregnancy, gestational diabetes mellitus, thyroid disease, intrahepatic cholestasis, appendicitis, and glomerulonephritis; and 70.0% (49/70) gave birth by cesarean section. The clinical characteristics were similar between the full analysis set and per protocol set (Table 1). Subsequent analyses were performed using the per-protocol set. Of the 94 infants, 52.1% (49/94) were twins; the median GA and birth weight were 32.9 (31.2, 34.0) weeks and 1770 (1477.5, 2042.5) g, respectively; the median NICU stay was 25.0 (16.0, 35.5) days; and the median age at discharge was 36.2 (35.4, 37.4) weeks of corrected age; the minimal and maximal age of mothers were 24 and 45, respectively.

**Table 1:** Clinical characteristics of mothers

	Full analysis set *n* = 94	Per-protocol set *n* = 70	*χ*^*2*^*/U*	df	p*
Age (year), median (IQR)	34.0 (31.0, 36.0)	34.0 (31.0, 36.0)	3190.50	N/A	0.74
Complications during pregnancy, n (%)	57 (60.6)	44 (62.9)	0.08	1	0.77
Cesarean section, n (%)	67 (71.3)	49 (70.0)	0.03	1	0.86
Gestational age (week), median (IQR)	32.6 (31.0, 34.1)	32.8 (30.9, 34.0)	3286.50	N/A	0.99
Primiparous, n (%)	61 (64.9)	45 (64.3)	0.006	1	0.94
Twin, n (%)	30 (31.9)	26 (37.1)	0.49	1	0.49

*Note.* Data are represented as number (%) or median (IQR, interquartile); * p-value is based on the results of a Mann-Whitney U test for continuous variables and a chi-square test for categorical one

### Lactation status

**Fig. 2:**
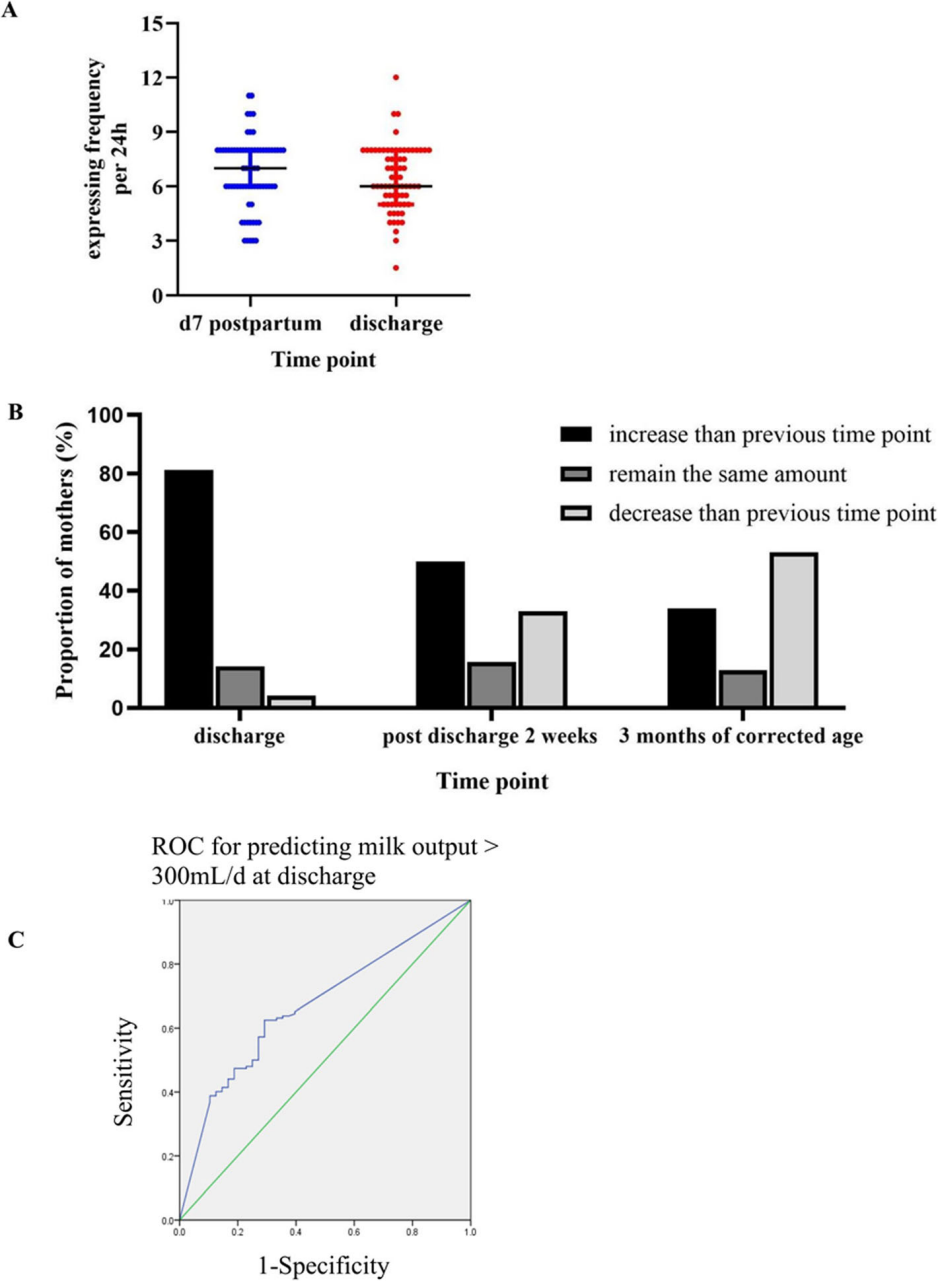
Expressing frequency and changes and predictive value of expressed milk volume. **A**. Expressing frequency on day 7 postpartum and discharge of infants. **B**. The proportions of mothers with increased, same, or decreased expressed milk volume compared with the previous time point. Expressed milk volume was measured on day 7 postpartum, at infant discharge, 2 weeks post-discharge, and 3 months corrected age. **C**. The predictive values of expressed milk volume on day 7 postpartum. ROC curve showed that expressed milk volume on day 7 postpartum reaching 190 mL/d was the optimal threshold for predicting expressed milk volume more than 300 mL/d at discharge (sensitivity, 72.5%; specificity, 73.7%; area under the curve [AUC], 0.77, 95% confidence interval, 0.65, 0.90; *p* = 0.001)

A survey of the types of breast pumps used by mothers showed that 65.7% (46/70), 32.9% (23/70), and 1.4% (1/70) of the mothers used unilateral electric pumps, bilateral electric pumps, and manual breast pumps, respectively. The median of expressing frequency per 24 h was 7.5 (5.5, 9.5) times and 6.0 (5.0, 8.0) times on day 7 postpartum and at infants’ discharge, respectively (Fig. 2A).

The median daily expressed milk volume was 220.0 (110.0, 400.0) mL on day 7 postpartum, which increased significantly to 525.0 (277.5, 762.5) mL at discharge (*U* = 1248.00, *p* < 0.001). On day 7 postpartum, 65.7% of the mothers expressed more milk than their infants’ uptake, and this proportion increased to 68.6% at the time of discharge. The proportions of mothers with increased expressed milk volume at discharge, 2 weeks post-discharge, and 3 months of corrected age compared with the previous time point were 81.4, 50.0, and 33.9%, respectively (Fig. 2B), indicating that fewer mothers had increased expressed milk volume and an increasing number of mothers produced less milk over time. At 3 months of corrected age, 53.2% of mothers produced less milk than at 2 weeks post-discharge.

The ROC analysis revealed that the expressed milk volume on day 7 postpartum of 190 mL/d was the optimal threshold for predicting expressed milk volume ≥ 300 mL/d at discharge (sensitivity, 72.5%; specificity, 73.7%; area under the curve [AUC], 0.77, 95% CI, 0.65, 0.90; *p* = 0.001, Fig. 2C).

Of the 70 mothers, 51.4% (36/70) and 20.0% (14/70) failed to achieve lactogenesis II within 4 and 7 days postpartum, respectively. Compared to mothers with normal lactogenesis II onset, mothers with delayed lactogenesis II onset, a higher proportion underwent cesarean section (80.6% vs. 58.8%, *χ*^*2*^ = 3.93, df = 1, *p* = 0.05) and a higher percentage were mothers giving first live birth (75.0% vs. 52.9%, *χ*^*2*^ = 3.71, df = 1, *p* = 0.05). Maternal age, complications during pregnancy, twin pregnancy, and gestational age were similar between the normal and delayed lactogenesis II onset groups (Table 2). Infants’ Apgar scores and mechanical ventilation durations were similar between the infants of mothers with normal and delayed lactogenesis II onset (Table 3).

**Table 2:** Comparison of the mothers’ characteristics and breastfeeding status of delayed versus normal lactogenesis ii onset groups

	Delayed group	Normal group	*χ*^*2*^*/U*	df	p*
**Mothers’ characteristic**	***n*** **= 36**	***n*** **= 34**			
Age years, median (IQR)	34.0 (31.0, 36.0)	34.0 (32.0, 36.0)	681.00	N/A	0.42
Complications during pregnancy, n (%)	26 (72.2)	18 (52.9)	2.78	1	0.10
Gestational age (week), Median (IQR)	32.4 (30.3, 33.7)	33.1 (31.2, 34.3)	775.00	N/A	0.06
Cesarean section, n (%)	29 (80.6)	20 (58.8)	3.93	1	**0.05**
First live birth, n (%)	27 (75.0)	18 (52.9)	3.71	1	**0.05**
Twin, n (%)	16 (44.4)	10 (29.4)	1.69	1	0.19
**Daily expressed milk volume (mL)**					
On postpartum day 7, median (IQR)	160.0 (88.1, 287.5)	300.0 (187.5, 550.0)	328.50	N/A	**0.001**
At discharge, median (IQR)	425.0 (200.0, 600.0)	612.5 (465.0, 850.0)	372.00	N/A	**0.005**

*Note.* Data are represented as number (%) or median (IQR, interquartile); * p-value is based on the results of a Mann-Whitney U test for continuous variables and a chi-square test for categorical one

**Table 3:** Comparison of the infants’ characteristics and breastfeeding status of mothers with delayed versus normal lactogenesis ii onset (*N* = 94)

	Delayed group (***n*** = 51)	Normal group (***n*** = 43)	*χ*^*2*^*/U*	df	p*
**Infants characteristic**					
Twin, n (%)	31 (60.8)	18 (41.9)	3.35	1	0.07
Apgar score at 5′<7, n (%)	1 (2.2)	0	–	–	–
Mechanical ventilation ≥3 days, n (%)	3 (5.9)	4 (9.3)	–	–	–
Length of stay, day, median (IQR)	25.0 (16.0, 31.0)	25.0 (15.0, 40.0)	1027.50	N/A	0.60
Age at discharge, weeks, median (IQR)	36.4 (35.6, 37.7)	36.1 (35.4, 37.0)	1175.00	N/A	0.55
**Breastfeeding status**					
Exclusive breastfeeding at discharge, n (%)	17 (33.3)	30 (69.8)	12.39	1	**0.001**
Breastfeeding at 3 months of corrected age, n (%)^1^	25 (55.6)	32 (88.9)	10.66	1	**0.001**
Exclusive breastfeeding at 3 months of corrected age, n (%)^1^	8 (17.8)	19 (52.8)	11.03	1	**0.001**

*Note.* Data are represented as number (%) or median (IQR, interquartile); * p-value is based on the results of a Mann-Whitney U test for continuous variables and a chi-square test for categorical one. IQR = interquartile

^1^At 3 months of corrected age, 62 mothers and 81 infants participated in the follow-up; of them, 32 mothers (with 45 infants) had delayed lactogenesis II onset and 30 mothers (with 36 infants) had normal onset

Multivariate logistic regression analysis revealed that older maternal age (adjusted odds ratio [aOR] = 1.19; 95% confidence interval [CI]: 1.01, 1.40), and first live birth (aOR = 4.81; 95% CI 1.43, 16.18) were significant independent predictors of delayed lactogenesis II onset (Table 4).

**Table 4:** Results of logistic regression analysis predicting delayed lactogenesis ii onset

Variables	aOR^1^	95%CI	p*
cesarean section	3.27	1.00, 10.68	0.05
older maternal age	1.19	1.01, 1.40	**0.03**
first live birth	4.81	1.43, 16.18	**0.01**
twins	1.96	0.64, 6.00	0.24

*Note.* Delayed lactogenesis II onset was included as dependent variable. A backward LR variable selection method was used to identify significant variables among cesarean section, maternal age, first live birth, pregnancy complications, and GA. The inclusion and removal of variables in models were based on p values of 0.05 and 0.10, respectively. Twin entered into the model through enter method due to potential clinical relevance. Cesarean section, older maternal age, first live birth, and twin entered the final model. The logistic regression model yielded statistical significance (*χ*^*2*^ = 14.98, df = 4, *p* = 0.005). The final model was checked by Hosmer-Lemeshow test for goodness-of-fit (*p* = 0.66)

* p-value is based on the results of a multivariate binary logistic regression analysis. aOR = adjusted Odds Ratio. CI = Confidence Interval

Mothers with delayed lactogenesis II onset had significantly lower daily expressed milk volume on day 7 postpartum (160.0 [88.1, 287.5] mL vs. 300.0 [187.5, 550.0] mL, *U* = 328.50, *p* = 0.001) and discharge day (425.0 [200.0, 600.0] vs. 612.5 [465.0, 850.0], *U* = 372.00, *p* = 0.005) than those mothers with normal onset (Table 2). In addition, the exclusive breastfeeding rate at discharge (33.3% vs. 69.8%, *χ*^*2*^ = 12.39, df = 1, *p* < 0.001), breastfeeding rate (55.6% vs. 88.9%, *χ*^*2*^ = 10.66, df = 1, *p* = 0.001) and exclusive breastfeeding rate (17.8% vs 52.8%, *χ*^*2*^ = 11.03, df = 1, *p* = 0.001) at 3 months of corrected age were also significantly lower in infants of mothers with delayed lactogenesis II onset (Table 3).

### Breastfeeding status at discharge and post-discharge

The proportions of infants receiving breastfeeding (70.4%) and exclusive breastfeeding (33.3%) at 3 months of corrected age were lower than those at discharge (breastfeeding, 78.7%; exclusive breastfeeding, 48.9%) and 2 weeks post-discharge (breastfeeding, 83.0%; exclusive breastfeeding, 48.9%, Table 5), although the differences were not statistically significant. At discharge, 21.3% (20/94) of the infants were fed with formula, and the most common reason for discontinuing breastfeeding was the infants’ clinical condition (14/20, Table 5). Among the 14 infants, one was diagnosed with cytomegalovirus (CMV) infection and 13 had gastrointestinal (GI) problems, including hematochezia or upper gastrointestinal bleeding (*n* = 6), abdominal distension (*n* = 5, one with both abdominal distension and hematochezia), diarrhea (*n* = 2), and necrotizing enterocolitis (*n* = 1), and 6 of the 13 infants resumed breastfeeding within 2 weeks after discharge. Four infants were diagnosed with necrotizing enterocolitis (Bell stage ≥IIA) during hospitalization, while three resumed breastfeeding after recovery, and the other was fed with an amino acid-based formula till 3 months of corrected age. At 2 weeks post-discharge, 17.0% (16/94) of infants discontinued breastfeeding, and the most common was also infant clinical condition (11/16, 10 cases of GI problems and 1 case of CMV infection). At 3 months of corrected age, 29.6% (24/81) of infants discontinued breastfeeding, and the most common reason was insufficient human milk (14/24, Table 5). There was a linear relationship between time and discontinued breastfeeding due to insufficient human milk (at discharge: 15.0%; at two weeks post-discharge: 31.3%; at 3 months of corrected age: 58.3%, *p* = 0.003) (Table 5).

**Table 5:** Breastfeeding status at discharge, 2 weeks post-discharge, and 3 months of corrected age

	Discharge, n (%)	Two weeks post-discharge, n (%)	3 months of corrected age, n (%)	*χ*^2^	df	p*
Mothers, *n*	70	70	62	–	–	–
Infants, *n*	94	94	81	–	–	–
Breastfeeding, n (%)	74 (78.7)	78 (83.0)	57 (70.4)	1.57	1	0.21
Exclusive breastfeeding, n (%)	46 (48.9)	46 (48.9)	27 (33.3)	4.07	1	**0.04**^**c**^
Formula feeding, n (%)	20 (21.3) ^a^	16 (17.0)	24 (29.6)	1.57	1	0.21
Reasons for discontinuing breastfeeding						
Infants’ factors	14 (14/20, 70.0)	11 (11/16, 68.8)	10 (10/24, 41.7)	3.71	1	0.05
Insufficient human milk	3 (3/20, 15.0)	5 (5/16, 31.3)	14 (14/24, 58.3)	8.80	1	**0.003**^**d**^
Others	3 (3/20, 15.0) ^b^	0	0	–	–	–

*Note.* Data are represented as number (%); * p-value is based on the results of chi-square test

^a^ Three infants were formula-fed during hospitalization because their mothers expressed very little milk and the families did not transport it to the hospital, and they started breastfeeding post-discharge

^b^ Three infants were breastfed during hospitalization, but their family did not transport milk to the hospital during the last three days of the infant hospital stay, but they all resumed breastfeeding post-discharge

^c^ R = 0.12, P (Approx. Sig.) = 0.04

^d^ R = -0.39, P (Approx. Sig.) = 0.002

We then investigated the factors that could be associated with continued breastfeeding at 3 months of corrected age. Of the 81 infants, 57 (70.4%) were breastfed and 24 (29.6%) were not at 3 months of corrected age (Table 5). Exclusive breastfeeding at discharge (59.6% vs. 25.0%, *p* = 0.004), infants receiving own mother’s milk ≥50% of total milk uptake at 2 weeks post-discharge (77.2% vs. 33.3%, *p <* 0.001), and direct feeding at 2 weeks post-discharge (70.2% vs. 37.5%, *p* = 0.006) were significantly associated with continued breastfeeding at 3 months of corrected age (Table 6). Multivariable logistic regression analysis revealed that twin was an independent predictor of discontinuing breastfeeding at 3 months of corrected age (aOR = 0.27; 95% CI 0.09, 0.86); meanwhile, in singleton infants, own mother’s milk ≥50% at 2 weeks post-discharge was an independent predictor of continued breastfeeding at 3 months of corrected age (aOR = 32.66; 95% CI 3.00, 355.25) (Table 7).

**Table 6:** Factors associated with continuous breastfeeding at 3 months of corrected age

	Breastfeeding	No breastfeeding	*χ*^*2*^	df	p*
	*n* = 57	*n* = 24			
Twin, n (%)	24 (42.1)	16 (66.7)	4.08	1	**0.04**
Breastfeeding at day 7, n (%)	39 (68.4)	14 (58.3)	0.76	1	0.38
Exclusive breastfeeding at day 7, n (%)	19 (33.3)	4 (16.7)	2.31	1	0.13
Breastfeeding at discharge, n (%)	46 (80.7)	16 (66.7)	1.85	1	0.17
Exclusive breastfeeding at discharge, n (%)	34 (59.6)	6 (25.0)	8.11	1	**0.004**
Own mother’s milk≥50% at 2 weeks post-discharge, n (%)	44 (77.2)	8 (33.3)	14.14	1	**< 0.001**
Direct feeding at 2 weeks post-discharge, n (%)	40 (70.2)	9 (37.5)	7.55	1	**0.006**

*Note.* Data are represented as number (%); * p-value is based on the results of chi-square test

**Table 7:** Results of logistic regression analysis predicting infants’ breastfeeding at 3 months of corrected age

Variables	All infants			Twins			Singleton		
	aOR^1^	95%CI	p*	aOR^1^	95%CI	p*	aOR^1^	95%CI	p*
Twin	0.27	0.09, 0.86	**0.03**	–	–	**–**	–	–	**–**
Gestational age							0.71	0.44, 1.12	0.14
Exclusive breastfeeding at discharge	–	–	**–**	7.29	1.74, 30.56	**0.007**	–	–	–
Own mother’s milk≥50% at 2 weeks post-discharge	8.20	2.6, −25.50	**< 0.001**	–	–	**–**	32.66	3.00, 355.25	**0.004**

*Note.* Breastfeeding at 3 months of corrected age was included as dependent variable. (1) In all infants, a backward LR variable selection method was used to identify significant variables among cesarean section, maternal age, twin, GA, exclusive breastfeeding at discharge, own mother’s milk ≥50% at 2 weeks post-discharge, and direct feeding at 2 weeks post-discharge. The inclusion and removal of variables in models were based on p values of 0.05 and 0.10, respectively. Twin and own mother’s milk ≥50% at 2 weeks post-discharge entered the model. The model yielded statistical significance (*χ*^*2*^ = 19.31, df = 2, *p <* 0.001), and was checked by Hosmer-Lemeshow test for goodness-of-fit (*p* = 0.52). (2) In twins, a backward LR variable selection method was used to identify significant variables among cesarean section, maternal age, GA, exclusive breastfeeding at discharge, own mother’s milk ≥50% at 2 weeks post-discharge, and direct feeding at 2 weeks post-discharge. However, the Hosmer-Lemeshow test *χ*^*2*^ = 0.00. (3) In singleton infants, a backward LR variable selection method was used to identify significant variables among cesarean section, maternal age, GA, exclusive breastfeeding at discharge, own mother’s milk ≥50% at 2 weeks post-discharge, and direct feeding at 2 weeks post-discharge. The inclusion and removal of variables in models were based on *p* values of 0.05 and 0.10, respectively. Gestational age and own mother’s milk ≥50% at 2 weeks post-discharge entered the model. The model yielded statistical significance (*χ*^*2*^ = 14.11, df = 2, *p =* 0.001), and was checked by Hosmer-Lemeshow test for goodness-of-fit (*p* = 0.47)

* p-value is based on the results of a multivariate binary logistic regression analysis. aOR = adjusted Odds Ratio. CI = Confidence Interval

### Challenges in continuing breastfeeding

A survey of the challenges faced by mothers in continuing breastfeeding found that difficulties in feeding their infants and low milk volume were the predominant challenges during the infant hospital stay. At 2 weeks post-discharge, feeding complications in infants and poor breastfeeding techniques became the predominant challenges. At 3 months of corrected age, difficulties in feeding infants and low milk volume were the top two challenges (Table 8).

**Table 8:** Challenges in breastfeeding during hospitalization and after post-discharge

	Challenges in breastfeeding	Response n (%)
**During hospital stay (*n*** = 70) *		
Associated with infants	Difficulties in feeding	22 (31.4)
	Feeding complications	5 (7.1)
	Breastfeeding discontinuation for diseases	3 (4.2)
Associated with mothers	Low human milk volume	13 (18.6)
	Lack of breastfeeding knowledge	9 (12.9)
	Difficulties in expressing milk	3 (4.2)
	Difficulties in milk transportation	2 (2.9)
**Post-discharge 2 weeks (*n*** **= 70)**		
Associated with infants	Feeding complications	20 (28.6)
	Difficulties in feeding	16 (22.9)
Associated with mothers	Poor breastfeeding techniques	17 (24.3)
	Low human milk volume	14 (20.0)
	Difficulties in expressing milk	3 (4.3)
	Lack of breastfeeding knowledge	4 (5.8)
**3 months of corrected age (*n*** **= 62)**		
Associated with infants	Difficulties in feeding	36 (58.1)
	Feeding complications	26 (41.9)
Associated with mothers	Low human milk volume	22 (35.5)
	Lack of breastfeeding knowledge	14 (24.7)
	Difficulties in expressing milk	6 (9.7)
	Lack of support from family	3 (4.8)
	Lack of support from the workplace	2 (3.2)

*Note.* Data are represented as number (%);

*Infants were fed mainly by health care staff during hospitalization, and by parents after discharge

## Discussion

### Lactogenesis II onset and breastfeeding

Previous studies have found that mothers giving birth to preterm infants experience difficult lactation, including delayed lactation [23], low expressed milk volume [14], and premature discontinuation of lactation [38]. In early lactation, one of the important indicators is lactogenesis II onset, which marks the time when mothers begin to produce copious amounts of milk [20].

Lactogenesis II usually occurs at 50–73 h postpartum, although the definition and measurement method of lactogenesis II onset may vary [20, 23]. In our study, mothers were discharged from hospital earlier than their infants and they expressed milk at home during their infant hospital stay. Thus, we defined the day when mothers were able to express 20 mL milk (the total amount from both sides) for three consecutive times as the day of lactogenesis II onset [41].

Delayed lactogenesis II is generally defined as a failure to initiate lactogenesis II within 72 h postpartum [42]. In this study, we found that 48.6% of the mothers of preterm infants achieved lactogenesis II within 4 days (72–96) postpartum, whereas the rest (51.4%) failed. Some studies have reported that lactogenesis II onset occurs at 97.15 h postpartum among mothers of preterm infants (infant birth weight < 1500 g) [43], and 36% of mothers of preterm infants (GA < 37 weeks) had delayed lactogenesis II onset (> 72 h postpartum) [26], which are similar to our results. Therefore, delayed lactogenesis II onset appears to be very common in mothers of preterm infants.

We found that older maternal age and first live birth were independent risk factors for delayed lactogenesis II onset, which is consistent with the results of previous studies [25, 42, 44–46]. First birth and unscheduled cesarean delivery might adversely affect lactogenesis and breastfeeding by increasing mothers’ stress [46].

In previous studies, early initiation and regular breast milk expression were found to be effective in reducing the risk of delayed lactogenesis II onset [47]. Therefore, in mothers of preterm infants who were of older maternal age and first live birth, providing education [48], and facilities (breast milk pump and lactation room) to improve early initiation and regular breast milk expression might be beneficial.

### Values predictive of expressed milk volume on day 7 postpartum

Our ROC analysis revealed that expressed milk volume > 190 mL/d on day 7 postpartum predicted expressed milk volume ≥ 300 mL/d at discharge. The result suggests that low expressed milk volume on day 7 postpartum might predict insufficient expressed milk volume later. Flacking et al. [49] found that insufficient expressed milk volume by the end of the first week postpartum was associated with a low breastfeeding rate in preterm infants at 2 months and 4 months postpartum. Notably, Omarsdottir et al. [36] used expressed milk volume at 2 weeks postpartum to predict breastfeeding status at 6 weeks postpartum and discharge. We used expressed milk volume on day 7 postpartum to predict expressed milk volume at discharge. Our method could effectively identify mothers with lactation difficulties at an earlier stage and thus guide healthcare professionals to intervene earlier to improve mothers’ lactation and breastfeeding.

### Breastfeeding status at 3 months of corrected age

In this study, at 3 months of corrected age, 70.4% of the infants were continued to be breastfed, and only 33.3% were breastfed exclusively. Meio et al. reported that breastfeeding and exclusive breastfeeding rates in preterm infants (GA < 33 weeks) at 3 months of corrected age were 62.4 and 4.3%, respectively [50], which is lower than our results. Continuous patient education in our institution and higher GA of preterm infants may contribute to the higher breastfeeding rate at 3 months of corrected age in this study.

Twins had no significant association with delayed lactogenesis II onset but were less likely to continue breastfeeding at 3 months of corrected age. The early cessation of breastfeeding in twins was conflicting in previous studies [8, 38]. The expressed milk volume in mothers of twins was higher than that of mothers of singleton infants (599 mL/day vs. 430 mL/day) [51], but less than 2 times of the latter one. As is shown in our study, insufficient human milk was the major reason for the discontinuation of breastfeeding at 3 months of corrected age. Therefore, insufficient breastmilk might be one of the difficulties in breastfeeding preterm twin infants. Further studies would be needed to investigate the specific difficulties in breastfeeding of mothers of preterm twin infants. The breastfeeding status and challenges of twin infants need more attention in the follow-up clinic.

At 3 months of corrected age, 53.2% of the mothers reported decreased milk production, a lower exclusive breastfeeding rate (33.3%) in infants, and a higher proportion of infants discontinuing breastfeeding because of insufficient human milk (58.3%), which is similar to the results of previous studies [38, 52].

In addition to mothers’ efforts, the overall health status and feeding tolerance of preterm infants also influence breastfeeding [53]. In this study, feeding was performed by healthcare professionals during their hospital stay. With the involvement of parents in feeding post-discharge, concerns and anxiety about feeding complications (abdominal distention, vomiting, choking, constipation, diarrhea, and hematochezia) increased significantly (28.6% vs.7.1%, *χ*^*2*^ = 10.96, df = 1, *p* = 0.001) and was maintained, which is consistent with the findings of Callen et al. [53].

Our study found that at 2 weeks post-discharge, the top challenge in breastfeeding associated with mothers was poor breastfeeding techniques. To improve breastfeeding skills, we recommend providing training on how to practice direct breastfeeding, use human milk fortifiers, adjust feeding volume, and clean breastfeeding devices. It is also important to train mothers on how to coordinate feeding rhythms with infants’ needs as described by Ikonen and Palmer [14, 54]. In addition, mothers should be instructed to recognize the signs of hunger and develop flexible feeding plans [55].

### Limitations

This was a single-center study. Although the number of participants was relatively small, the management and education of the mothers were highly consistent in this study. The GA range of the participating preterm infants was wide. Twins were also included in the analysis. The proportion of twins in this study is higher than that in some studies [56] but is consistent with our recent studies in the same institution (23.3–38.0%) [22, 57] and similar to some other studies in NICUs in China (24.7–34.0%) [16, 58, 59] and other countries (31.8–40.5%) [9, 38]. The inclusion of twins may lead to a lower rate of exclusive breastfeeding than in other studies. We did not investigate the difference in challenges faced by mothers of twins and singleton infants. The study did not include information on mothers’ long-term use of medication and during labor [9, 24] and socioeconomic status [36], which may be associated with breastfeeding and exclusive breastfeeding in preterm infants. Follow-up after 3 months of corrected age might further elucidate the breastfeeding status of mothers of preterm infants. In addition, in this study, frozen expressed milk was used to feed the infants. We did not investigate how frozen expressed milk could influence breastfeeding duration and the proportion of human milk in infants’ diets.

Regardless of these limitations, our study provides information for the early prediction of breastfeeding status which may help provide better-targeted instructions to the families of preterm infants. In addition, we chose the time points of 7 days postpartum, at infants’ discharge, 2 weeks post-discharge, and 3 months of corrected age to ask the mothers about their breastfeeding status and difficulties. These time points could best present the breastfeeding status during mother-infant separation, during the adaptation after discharge, and relatively long-term after discharge. Our time points may be better suited for preterm infants with different gestational ages, and better demonstrate the difficulties parents encounter after infants’ discharge.

## Conclusions

Older maternal age, and first live birth were independent predictors of delayed lactogenesis II. Milk output volume on day 7 postpartum > 190 mL/d predicted ≥300 mL/d milk output at discharge. Difficulties in breastfeeding infants, poor breastfeeding techniques, and insufficient milk output were the predominant challenges faced by mothers. Thus, interventions targeting the improvement of early postpartum lactation and training targeting to improve breastfeeding techniques and reduce the difficulties in breastfeeding may increase breastfeeding adoption in mothers giving birth to preterm infants.

## Supplementary Information

**Additional file 1:** Questionnaires on day 7 postpartum, at discharge, at 2 weeks post-discharge, and 3 months of corrected age (translated in English).

## Data Availability

The datasets generated and/or analyzed during the current study are not publicly available for ethical reasons but are available from the corresponding author upon reasonable request.

## Abbreviations

NICU: neonatal intensive care unit
GA: gestational age
HMF: human milk fortifier
ROC: receiver operating characteristic
