# Supplementary material for: A prospective cohort study on lactation status and breastfeeding challenges in mothers giving birth to preterm infants
Source: Int Breastfeed J. 2022 Jan 10;17:6. doi: 10.1186/s13006-021-00447-4 (PMC8751123; doi:10.1186/s13006-021-00447-4)
Supplement: Supplementary file 1 — Additional file 1: Questionnaires on day 7 postpartum, at discharge, at 2 weeks post-discharge, and 3 months of corrected age (translated in English). [file 13006_2021_447_MOESM1_ESM.docx]

**Additional File 1. Interview Questions**

| **The questionnaire on day 7 postpartum** |
| --- |
| 1. Did you send expressed milk for your baby in NICU? 2. Yes 3. No. The reason was: a. transient b. permanent. 4. How do you express milk? 5. With a manual breast pump. 6. With a unilateral electric pump. 7. With a bilateral electric pump. 8. How often did you express milk in the past 24 hours? _____ times/day 9. Did you express milk at night? 10. Yes. Every ______ hours. 11. No. 12. How much milk did you express in the past 24 hours? ______ml. 13. When did you first able to express 20mL milk (the total amount from both sides) for 3 consecutive times? ____(mm)_____(dd). |

| **The questionnaire at discharge** |
| --- |
| 1. How often did you express milk in the past 24 hours? _____ times/day 2. Did you express milk at night? 3. Yes. Every ______ hours. 4. No. 5. How much milk did you express for a day on average in the past 3 days? ______ml. 6. Did the amount of expressed milk increase or decrease than day 7 postpartum? 7. Increased. 8. Remained the same. 9. Decreased. 10. How much expressed milk stored in your home (count as 100ml/package)? 11. None. 12. <10 packages. 13. 10-29 packages. 14. 30-49 packages. 15. 50-99 packages. 16. ≥100 packages. 17. What are your main concerns and barriers to breastfeeding during your baby’s hospital stay? |

| **The questionnaire at 2 weeks post-discharge** |
| --- |
| 1. What is the milk your baby fed with at present? 2. Human milk:   a. exclusive breastfeeding. b. more than half of the milk. c. less than half of the milk. d. no human milk.   1. Formula: a. use formula ____ times/ day. b. no formula. 2. Human milk fortifier (HMF): a. add HMF. b. no HMF. 3. How do you feed your baby at present? 4. Exclusive breastfeeding:   a. direct feeding. b. indirect feeding (feeding expressed milk with a bottle or spoon). c. both direct and indirect feeding.   1. Mixed feeding. 2. Formula 3. How much do you feed your baby? ____ml, _____times/ day. 4. If you already discontinued breastfeeding, the reason was: 5. I had to take a medicine that was not advisable during breastfeeding. 6. I had a disease that was not advisable for breastfeeding. 7. Low expressed milk volume early postpartum. 8. Low milk production after discharge. 9. Worried about the inadequate nutrition in my milk. 10. Unfamiliar with feeding knowledge and skills. 11. The process of expressing, storage, heating was too cumbersome. 12. Worried about the incomplete disinfection during the storage and heating process, which might lead to infection of my baby. 13. Lack of family support for breastfeeding. 14. Family members’ objection to breastfeeding. 15. My baby had poor weight gain, diarrhea, or bloody stool during breastfeeding. 16. Economic reasons. 17. Others. ___________ 18. Did you feel the expressed milk increase or decrease after your baby came home? 19. Increased. 20. Remain the same as discharge. 21. Decreased. 22. Don’t know. 23. Have you tried direct feeding? 24. Yes. 25. No. 26. After your baby’s discharge, what are your main concerns and barriers to breastfeeding? |

| **The questionnaire at 3 months of corrected age** |
| --- |
| 1. What is the milk your baby fed with at present?   1. Human milk: a. exclusive breastfeeding. b. more than half of the milk. c. less than half of the milk. d. no human milk. 2. Formula: a. use formula ____ times/ day. b. no formula. 3. Human milk fortifier (HMF): a. add HMF. b. no HMF. 4. How do you feed your baby at present? 5. Exclusive breastfeeding: a. direct feeding. b. indirect feeding (feeding expressed milk with a bottle or spoon). c. both direct and indirect feeding. 6. Mixed feeding. 7. Formula feeding. 8. How much do you feed your baby? ____ml, _____times/ day. 9. Did you feel the expressed milk increase or decrease after your baby came home? 10. Increased. 11. Remain the same as discharge. 12. Decreased. 13. Don’t know. 14. Have you tried direct feeding? 15. Yes. 16. No. 17. If you already discontinued breastfeeding, the reason was: 18. My baby had poor weight gain. 19. My baby had to take a special medical formula for health reasons, like: a. eczema b. abdominal distension c. bloody stool d. diarrhea e.______ 20. I don’t have enough time to feed my baby /express milk for my baby after maternal leave. 21. There is no lactation room in my workplace to express milk. 22. There is no refrigerator in my workplace to store expressed milk. 23. It is inconvenient to bring a pump to my workplace. 24. Discontinuation of lactation. 25. I had to take a medicine that was not advisable during breastfeeding. 26. I had a disease that was not advisable for breastfeeding. 27. Lack of family support. 28. I don’t have enough time because I have to take care of other kids. 29. I don’t think breastfeeding is important. 30. The process of expressing, storage, heating was too cumbersome. 31. Worried about the incomplete disinfection during the storage and heating process, which might lead to infection of my baby. 32. Others. ___________ 33. From baby’s 2 weeks post-discharge to 3 months of corrected age, what are your main concerns and barriers in breastfeeding? |
